# PECAM-Independent Thioglycollate Peritonitis Is Associated With a Locus on Murine Chromosome 2

**DOI:** 10.1371/journal.pone.0004316

**Published:** 2009-01-30

**Authors:** Michael A. Seidman, Tina W. Chew, Alan R. Schenkel, William A. Muller

**Affiliations:** 1 Department of Pathology, Weill Cornell Medical College, New York, New York, United States of America; 2 Department of Microbiology, Immunology, and Pathology, College of Veterinary Medicine and Biological Science, Colorado State University, Fort Collins, Colorado, United States of America; Massachusetts General Hospital/Harvard University, United States of America

## Abstract

**Background:**

Previous studies have demonstrated that knockout or inhibition of Platelet/Endothelial Cell Adhesion Molecule (PECAM, CD31) in a number of murine strains results in impaired inflammatory responses, but that no such phenotype is seen in the C57BL/6 (B6) murine background.

**Methodology/Principal Findings:**

We have undertaken a quantitative trait locus (QTL) mapping effort between FVB/n (FVB) and B6 mice deficient for PECAM to identify the gene or genes responsible for this unique feature of B6 mice. We have identified a locus on murine chromosome 2 at approximately 35.8 Mb that is strongly associated (LOD score = 9.0) with inflammatory responses in the absence of PECAM.

**Conclusions/Significance:**

These data potentiate further study of the diapedesis machinery, as well as potential identification of new components of this machinery. As such, this study is an important step to better understanding the processes of inflammation.

## Introduction

Inflammation necessitates that leukocytes leave the blood stream where they circulate and enter into tissue to engage in effector functions. A well developed series of steps is involved regulating the exit of leukocytes from the blood stream across the endothelial barrier, a process referred to as leukocyte transendothelial migration (TEM) [1], [2]. The committed step of this process is referred to as diapedesis, the step where the leukocyte actually squeezes between two endothelial cells. It has been shown that the Platelet/Endothelial Cell Adhesion Molecule (PECAM, CD31) is integral to diapedesis. When the PECAM knockout mouse was first made, however, no apparent blockade of leukocyte TEM was observed in several *in vivo* models [3]. This early model was developed in a hybrid of 129/J and C57BL/6 (B6) mice, and was further studied in mice backcrossed extensively onto the B6 background. While a delay was observed in the time it took leukocytes to cross the basement membrane, and a number of other subtle defects have been described in the literature, the expected blockade of diapedesis was not seen [3], [4], [5], [6], [7], [8], [9], [10], [11], [12], [13].

Subsequent studies observed that transgenic FVB/n (FVB) mice constitutively expressing moderate levels of a soluble “decoy” PECAM-Fc chimera in their circulation did manifest a blockade in TEM of monocytes and neutrophils [14]. Studies were undertaken that established that soluble PECAM-Fc chimeras and antibodies against PECAM could block normal TEM of monocytes and neutrophils in live mice for all strains tested except the B6 strain [15]. When the PECAM knockout was backcrossed into the FVB background, a blockade in TEM was, in fact, observed [15]. These data suggested an apparently unique ability of the B6 mice to disregard loss or functional blockade of PECAM with respect to TEM.

Given the inbred nature of laboratory mouse strains, it stands to reason that a unique genetic element (or elements) in the B6 strain is responsible for the observed phenomenon of PECAM-independent TEM. As identification of such genetic elements could reveal genes encoding for previously unidentified components of the diapedesis machinery, and thus provide insights into the exact mechanisms of this machinery, we undertook an effort to map these genetic differences.

The technique of quantitative trait locus (QTL) mapping is a powerful form of genetic linage mapping that can be used to dissect such phenotypes where a quantitative difference exists between genetically inbred mouse strains [16]. This particular technique relies on comparing the quantitative phenotypic results for a number of mice resulting from the breeding of two different mouse strains with distinct quantitative differences in a given phenotype (e.g. height, weight, serum cholesterol values, etc.). For each mouse in the offspring, typically of the F_2_ (second filial, i.e. offspring of the first filial generation, which are mice generated by crossing the two parental strains) or B_2_ (backcross; offspring of mating mice from the first filial generation to mice from one parental strain) generation, a quantitative value for the phenotype of interest is obtained. These values are compared to the genotype of specific markers throughout the offspring's genomes that are known to be different between the parental strains, and thus indicating the parental strain of origin for each marker region, and thus also each gene linked to that region, throughout those genomes. Typically, due to the development of appropriate high-throughput assays, the variations mapped in such efforts are either microsatellite repeat markers, where variation is determined by different lengths of the microsatellite between parent strains assayed via multiplex PCR, or single-nucleotide polymorphisms (SNPs), where the parent strains are known to have different alleles for the given SNP assayed through microarray technologies. Computer software is then utilized to perform a regression analysis at each possible locus compared to the quantitative phenotype results, often taking into account linkage of nearby loci, and returning a likelihood of each given locus being associated with the phenotype of interest.

Utilizing a previously described microsatellite panel known to distinguish between genetic elements in FVB and B6 mice [17], as well as a well-characterized SNP database capable of similar differentiation between the FVB and B6 murine strains [18], we undertook a QTL mapping strategy to identify the locus or loci of genetic trait or traits responsible for the apparently unique ability of leukocytes from B6 mice, as compared primarily to those from FVB mice, to transmigrate even in the absence of PECAM.

## Materials and Methods

### Mice

All animal procedures were reviewed and approved by the Institutional Use and Care of Animals Committee (IACUC) of Weill Cornell Medical College. C57BL/6 *Pecam* ^−/−^ and FVB/n *Pecam* ^−/−^ mice were developed as previously described with at least nine backcrosses into each respective strain [3], [15]. Wild-type C57BL/6J (“B6”), C57BL/10J (“B10”), DBA/2J (“D2”), FVB/nChr (“FVB”), Balb/cChr (“Balb/c”) mice were purchased from either The Jackson Laboratory (Bar Harbor, ME), or Charles River Laboratories (Wilmington, MA), as indicated by the strain name suffix. All mice were maintained and bred at the Weill Cornell Medical College Research Animal Resource Center.

### Thioglycollate Peritonitis

Thioglycollate peritonitis (TGP) was performed as described previously [3], [14], [15], [19] and in accordance with the policies set forth by the Institutional Animal Care and Use Committee (IACUC) of Weill Cornell Medical College. Wild-type mice were injected via tail vein with 100 µL of either PBS or a solution of anti-PECAM (Armenian hamster anti-mouse, clone 2H8, [20]) or soluble PECAM-Fc chimera [14], [15] in PBS (both blocking reagents at 1 mg/mL); prior studies utilizing isotype control antibodies revealed no difference from PBS-only injection [20], [21]. One hour later (or at the start of the experiment, in the case of knockout mice), mice were injected intraperitoneally with 1 mL of 4% Difco thioglycollate broth (Becton-Dickinson, Franklin Lakes, NJ). After 18 hr, mice were euthanized and a peritoneal lavage was performed using 5 mL of HBSS containing 10 mM ethylene diamine tetraacetic acid (EDTA). Lavage material was assayed manually for total (hemacytometer) and differential cell counts (cytospins stained with modified Wright-Giemsa stain, Protocol Hema3, Fisher Diagnostics, Middletown, VA). Murine blood was also collected to ensure comparable peripheral blood counts between groups. Data were tabulated and analyzed using Microsoft Excel (Microsoft Corporation, Redmond, WA) and the R statistical software package (http://cran.r-project.org/).

### Low-density QTL mapping and analysis

Kidneys from mice used in the thioglycollate peritonitis were removed immediately following peritoneal lavage and stored at −80°C. From these kidneys, genomic DNA was extracted using the Qiagen DNeasy Blood and Tissue Kit (Qiagen Inc., Valencia, CA) as described in the manufacturer's protocol. Genomic DNA was analyzed for Whitehead Institute microsatellite repeat polymorphisms using the protocol described by Teupser *et al.* [17] to distinguish between FVB and B6 mice. Assays were performed by the Rockefeller University Genomics Resource Center, New York, NY, using multiplex PCR with fluorescent dye-coupled primers and laser scanning capillary gel electrophoresis. Resulting genotype data were tabulated in Microsoft Excel and then analyzed using the R/qtl package [22].

### Medium-density QTL mapping and analysis

DNA from the same mice was subjected to additional genotyping using the Illumina Mouse MD (Medium Density) Linkage Panel (Illumina, Inc., San Diego, CA) [18], performed by the Rockefeller University Genomics Resource Center. Resulting data were analyzed manually within the region of interest, and genome-wide using the R/qtl software package.

### Gene database searches

Genes in the area(s) of interest were identified using databases at the NCBI (http://www.ncbi.nlm.nih.gov/), UCSC Genome Browser (http://genome.ucsc.edu/), Ensembl (http://www.ensembl.org/), and Mouse Genome Informatics (MGI, http://www.informatics.jax.org/). Map positions are given in NCBI sequence positions.

## Results

### *Pecam^−/−^* mouse breeding

SNP genotyping, using the Illumina medium density array, of the parental B6 and FVB PECAM-deficient mice revealed the B6 mice to be 98.2% backcrossed and the FVB mice to be 99.7% backcrossed relative to standard stock mice of the respective strain. These values are within conventional standards for use of knockout mice.

### Response to inflammatory challenge

**Figure 1:**
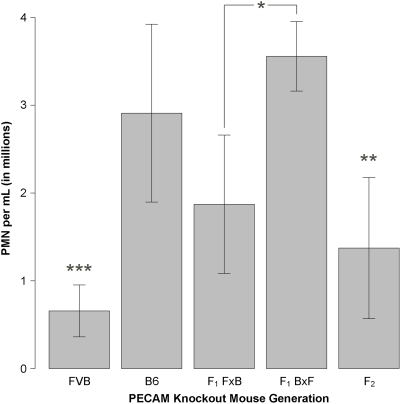
Thioglycollate peritonitis in three generations of PECAM-deficient mice. Thioglycollate peritonitis (TGP) was performed as described in Materials and Methods. All bars show mice that received 1 mL of 4% thioglycollate broth intraperitoneally. The mean inflammatory score and standard error of that mean is shown for each generation of knockout mice generated during the crossing of FVB and B6 PECAM-deficient mice. Data include 14 FVB mice, 14 B6 mice, 6 F_1_ BxF mice, 7 F_1_ FxB mice, and 110 F_2_ mice. *: groups vary from each other significantly; **: group varies from several other groups significantly; ***: group varies from all other groups significantly; detailed statistical comparisons are reported in Supplemental Table S1.

**Figure 2:**
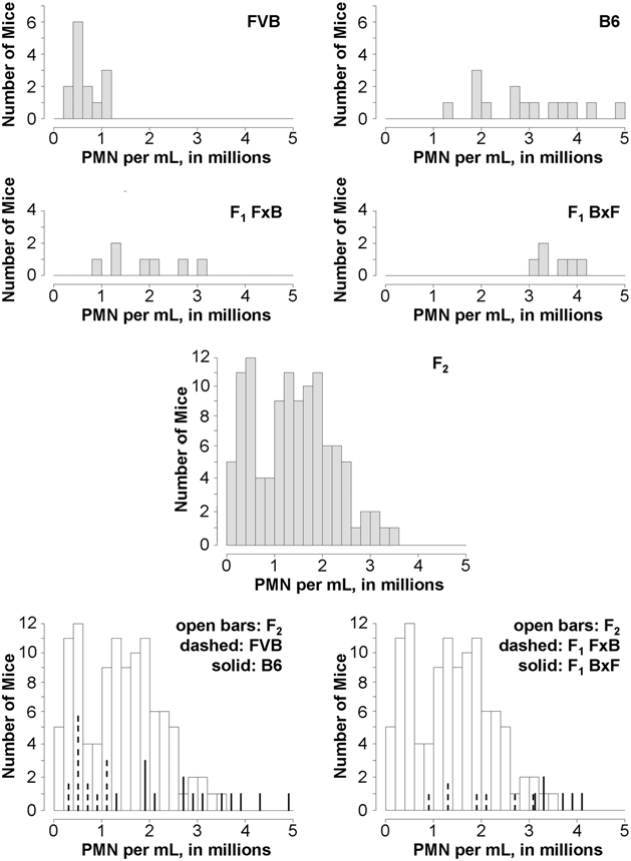
Histograms showing distributions of individuals within three generations of PECAM-deficient mice. The histograms each show the number of mice having inflammation scores within a given range (cell increment 2×10^5^ PMN per mL). Overlays allow for visualization of the degree of overlap between the respective populations.

F_1_ mice generated from crosses of B6 and FVB PECAM-deficient mice showed a phenotype most consistent with the B6 parents, i.e. they tended to display ongoing robust inflammatory responses to thioglycollate despite lack of PECAM (Figures 1 and 2; Supplemental Table S1). There was an equivalent number of male and female offspring in the F_1_ generation with no significant difference between them in terms of thioglycollate response (data not shown). There was a difference observed, however, between the mice generated from B6 mothers and FVB fathers (BxF) as compared to those from FVB mothers and B6 fathers (FxB), suggesting an imprinted trait modifying the F_1_ phenotypes. Both groups varied significantly from FVB parents and not from B6 parents, suggesting a dominantly inherited trait, but those with B6 mothers showed more robust responses than those mice with FVB mothers. At this time, we have not performed any studies to attempt to identify the imprinted gene or genes involved in this phenomenon.

F_2_ mice generated from the F_1_ mice showed a distribution of inflammatory responses extending between the two parental strains (Figures 1 and 2). The mean response varied from all other groups except the F_1_ FxB mice (Supplemental Table S1). There was no significant correlation between the response to thioglycollate and the gender (Supplemental Figure S1), coat color (Supplemental Figure S2), or age (Supplemental Figure S3) of these mice. Analysis of the distribution of the inflammatory phenotypes in the F_2_ mice was conducted using statistical modeling (Supplemental Figure S4). These models are consistent with a single-gene Mendelian model having a classic 1∶3 distribution, and thus these data are consistent with a single gene autosomal dominant Mendelian trait.

### Low-density QTL mapping

**Figure 3:**
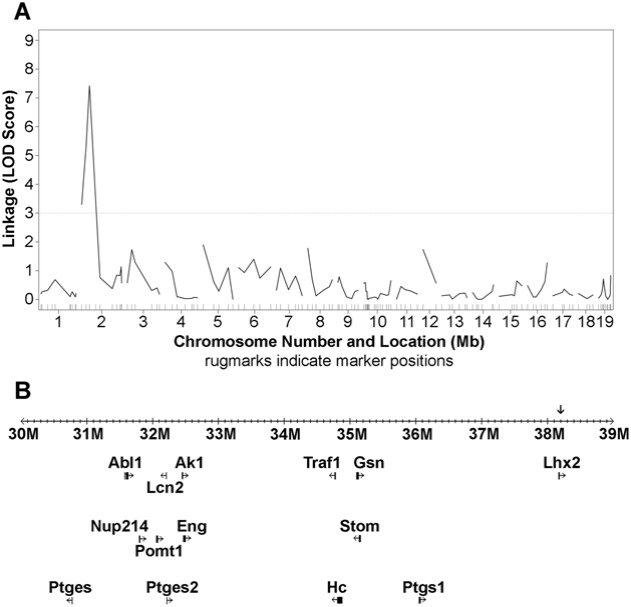
Low-density QTL mapping results. The low-density microsatellite mapping study revealed a single locus (panel A) breaking genome-wide association cutoffs. Panel B shows a close-up view of that locus, at 38.2 Mb (NCBI map coordinates), indicated by an arrow, as well as a number of nearby genes identified in databases as relevant to inflammation, immunity, or hematologic function.

To identify the gene responsible for the phenotypes we observed, we undertook QTL mapping in the F_2_ mice. An initial mapping study using approximately 150 markers distributed throughout the murine genome identified a single significant locus on the proximal end of chromosome 2 (Figure 3A; peak at d2mit7, 38.2 Mb). Statistical significance for such loci is traditionally measured by a logarithm of odds, or LOD, score, which is roughly equivalent to negative logarithm of the p-value for the significance of association; i.e. a locus with LOD = 4 is 10-times more strongly associated to the phenotype of interest than is one with LOD = 3. Statistical significance is typically reached at a value of LOD = 3.0; this locus had a LOD score of 7.5, suggesting very strong association between the locus and the phenotype of interest.

Several of the genes near this locus are identified in the schematic map in Figure 3B. These genes were identified using the Mouse Genomics Informatics site's search engine to identify genes associated with immunologic or hematologic function, including the Abl1 (c-abl) oncogene, Traf1 (TNF-receptor associated factor 1), Eng (endoglin), the prostaglandin synthases Ptgs1/COX-1, Ptges1, and Ptges2, and the hemolytic complement gene Hc (C5).

### Medium-density QTL mapping

**Figure 4:**
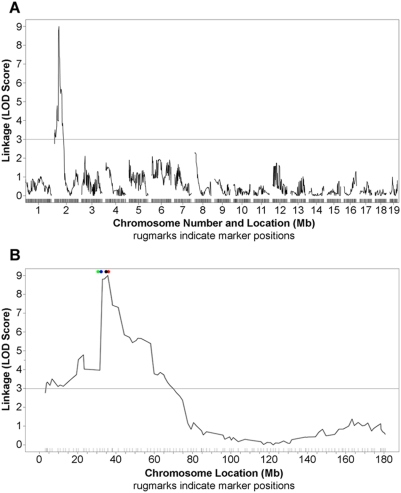
Medium-density QTL mapping results. Using a proprietary allele-specific primer extension based assay, the Illumina GoldenGate technology, the QTL peak was narrowed to a point at 35.8 Mb (panel A). Near this well-defined peak are four genes of particular interest (panel B, colored circles at top of graph, from left to right): *Ptges*, coding for mPGES-1 (prostaglandin E_2_ synthase 1, green); *Ptges2*, coding for PGES-2 (prostaglandin E_2_ synthase 2, blue); *Hc*, coding for hemolytic complement component C5 (black); and *Ptgs1*, coding for the protein commonly known as COX-1 (red); these four genes are also indicated in the lower row of genes identified in Figure 3B. The LOD score = 3.0 threshhold for significance is inidcated by a horizontal grey line in both panels.

We then undertook a medium-density mapping study using the Illumina Medium Density Mouse Linkage Analysis system [18]. We chose to map only those mice with known crossover events in the region of interest, and we successfully obtained medium-density genotypes from fourteen of these mice, as well as one mouse from each parental strain. This medium-density mapping study supported the association of the identified QTL peak with the phenotype of interest, and furthermore strengthened the LOD score at the QTL peak (Figure 4A, LOD = 9.0) and narrowed the region of interest (Figure 4B, peak 35.8 Mb).

### Analysis of *Hc* as a candidate gene

**Figure 5:**
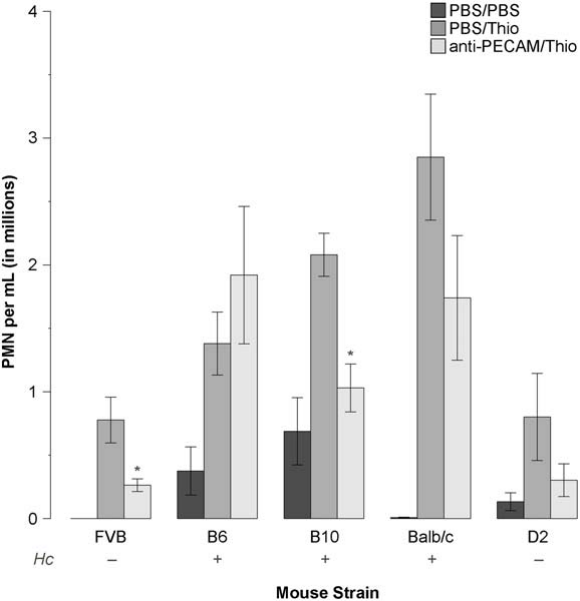
Comparison of mouse strain C5 status vs. response to PECAM-blockade in thioglycollate peritonitis (TGP). TGP was performed as described in Materials and Methods. Treatment groups shown include PBS both intravenously and intraperitoneally (PBS/PBS), PBS intravenously followed by 1 mL of 4% thioglycollate broth intraperitoneally (PBS/Thio), and 100 µg of anti-PECAM (clone 2H8) intravenously followed by 1 mL of 4% thioglycollate broth intraperitoneally (anti-PECAM/Thio). The graph shows data from a single representative experiment, normalized to the FVB thioglycollate-stimulated condition. The mean normalized inflammatory score and standard error of that mean are plotted for each of five mouse strains treated with PBS intravenously and intraperitoneally (PBS/PBS, dark bars), PBS intravenously followed by thioglycollate broth intraperitoneally (PBS/Thio, medium-density gray bars), or anti-PECAM clone 2H8 intravenously followed by thioglycollate broth intraperitoneally (anti-PECAM/Thio, light gray bars). Three strains, B6, B10, and Balb/c, are C5-sufficient, while FVB and D2 are C5-deficient. Significant blockades are indicated by an asterisk. The blockade p-values (i.e. *t*-test between thioglycollate and anti-PECAM plus thioglycollate, two-tailed, unequal variance) are as follows: FVB: p = 0.045; B6: p = 0.407; B10: p = 0.003; Balb/c: p = 0.149; D2: p = 0.231. B10: C57BL/10 mice; D2: DBA/2 mice.

Looking at the literature for published QTLs in the region near murine Chr 2, 35.8 Mb, a majority of the immunological phenotypes are associated with the hemolytic complement gene, *Hc*, a.k.a. complement component C5 [23], [24], [25], [26], [27], [28], [29], [30]. Since this gene is known to be a major variant in inbred mouse populations [31], [32], [33], [34], [35], with some strains carrying a premature stop codon and thus effectively being *Hc*-null, and further recognizing B6 mice as *Hc*-positive and FVB as *Hc*-null, we decided to see if other mouse strains demonstrated the PECAM-independent inflammatory phenotype in correlation with their *Hc* status. In conjunction with previously published data [15], we found that the thioglycollate peritonitis results were independent of *Hc*-status; only B6 mice demonstrate PECAM-independent thioglycollate peritonitis (Figure 5). It is notable that while the blockade in Balb/c and DBA/2 mice does not reach statistical significance, the B10 mouse strain does clearly show a blockade, and yet is *Hc*-positive.

## Discussion

We have identified a single locus, at 35.8 Mb on murine chromosome 2, associated with PECAM-independent inflammation in the thioglycollate peritonitis (TGP) model. This trait is inherited in an autosomal dominant Mendelian manner, although other traits, particularly an imprinted trait observed in the F_1_ mice, do modify the observed phenotypes. We suggest a name of *Pitgp* for this locus, short for **P**ECAM-**i**ndependent **t**hio**g**lycollate **p**eritonitis.

While a number of existing QTLs are near this same locus and associated with the hemolytic complement component C5 *Hc* gene, it appears that the classic variation in this gene, a premature stop codon, does not account for the variation seen, as only B6 mice show PECAM-independent TGP, while other *Hc*-positive strains do not.

The other gene candidates at the QTL locus primarily include COX-1 (*Ptgs1*), and the PGE_2_-synthases 1 and 2 (*Ptges* and *Ptges2*). Prostaglandins, especially prostaglandin E_2_, are known to be potent mediators of inflammatory responses *in vivo*, and it would seem reasonable that the prostaglandin synthetic enzymes may play a role in regulating TEM. Unfortunately, due to the complex biology of prostaglandins [36], [37], [38], [39], [40], [41], [42], we have not yet been able to evaluate these candidates functionally *in vivo*. Knockout mice are available for the genes in question [43], [44], but many generations of backcrossing or, to avoid carryover of linked loci, *de novo* creation of knockouts in the appropriate strains will be required before we can evaluate the function of these genes in the context of this study. Further genetic dissection of this locus is limited by the power of linkage analysis and the mosaic structure of the murine genome, leading to a high degree of linkage disequilibrium in the region of interest [45]. While the development of congenic mice for these loci and appropriate strains of candidate gene knockouts are under development, it will be some time before the definitive gene or genes responsible for the phenotype associated with *Pitgp1* locus is/are identified.

It is likely that the *Pitgp* ^B6^ allele is a gain-of-function mutation that confers some novel adhesion and/or signaling pathway onto B6 mice not found in other strains. However, it should be noted that in no strain of mouse does PECAM blockade or genetic deletion lead to overt immunosuppression. As such, there must be competent immune mechanisms in all mouse strains even in the absence of PECAM. It becomes necessary, therefore, to ask which circumstances require the use of PECAM in TEM and which are independent. A number of other inflammatory models have been examined using PECAM blockade or knockout, some being PECAM-dependent while others are PECAM-independent. Many of the situations where TEM is PECAM-independent are conditions where direct activation of the leukocyte may have taken place [46]. It is possible, therefore, that factors released by activated leukocytes obviate the need for PECAM-PECAM engagement, and the gain-of-function mutation mapped in this study causes dysregulated release of such factors.

Regardless of the mechanism at work, the identification of this locus allows for breeding of congenic mice for the *Pitgp* allele onto various genetic backgrounds. Using these mice, we hope to explore the full range of inflammatory stimuli for which this QTL explains interstrain differences. In combination with the data showing which events are PECAM-dependent, it becomes possible to begin building a more complete picture of the mechanisms leading to TEM in any given setting. Additionally, once we have the murine tools to follow the effect of both the PECAM and *Pitgp* loci, we may be able to identify new loci that play a role in other strains or other inflammatory settings, and thus possibly identify yet more signals that play a role in the control of TEM.

## Supporting Information

Table S1 P-values from pairwise statistical comparisons of the data presented in Figure 1. Significant values are indicated in bold.
(0.02 MB XLS)

Figure S1 Histograms comparing the distribution of female and male mice in the F_2_ generation. The histograms each show the number of mice of the given gender having inflammation scores within a given range (cell increment 2×10^5^ PMN per mL). Statistical comparison of the two groups yields a Welch two-sample t-test p-value of 0.296 and a Mann-Whitney (Wilcoxon) u-test p-value of 0.283.
(6.63 MB TIF)

Figure S2 Histograms comparing the distribution of black, brown, and white mice in the F_2_ generation. The histograms each show the number of mice of the given coat color having inflammation scores within a given range (cell increment 2×10^5^ PMN per mL). Statistical comparison of the black and brown groups yields a Welch two-sample t-test p-value of 0.750 and a Mann-Whitney (Wilcoxon) u-test p-value of 0.924. Statistical comparison of the black and white groups yields a Welch two-sample t-test p-value of 0.515 and a Mann-Whitney (Wilcoxon) u-test p-value of 0.386. Statistical comparison of the brown and white groups yields a Welch two-sample t-test p-value of 0.225 and a Mann-Whitney (Wilcoxon) u-test p-value of 0.204.
(8.49 MB TIF)

Figure S3 Scatterplot comparing the inflammation scores mice in the F_2_ generation to the age of the mouse at the time of the experiment. Each circle on the plot represents a single mouse. The dashed line represents a least-squares regression fit; R^2^ value = 0.002, p-value 0.398.
(10.74 MB TIF)

Figure S4 Statistical modelling of the F2 population. Statistical models representing the mathematical distribution of the F2 mice were built in the R statistical package to evaluate for the number of subpopulation that could be identified. Non-parametric densities of the various distributions were estimated using three different functions, density, bkde (at two different kernel values), and hist. The four resulting probability estimates were modeled with the non-linear modeling function, nls, using varying numbers of normally distributed subpopulations. Coefficients were tested against expectations using chi-square and Fisher's exact tests (both tests designed to compare expected and observed count data), and model errors were the residual summed square values (an algorithm that computes the difference between the model and the actual data at hundreds of data points, and returns an unsigned sum of the differences). A representative non-parametric density model is shown overlaying the parental, F_1_, and F_2_ populations in panels A and B. Panel C shows the resulting bimodal and unimodal mathematical models, demonstrating that a bimodal distribution, i.e. a mathematical model composed of two overlapping but distinct subpopulations, is more robust than a unimodal model, i.e. a mathematical model in which the mice all appear to be part of a single large population. Trimodal models, not shown, were only subtle variations on the bimodal models, rather than a distinctly different distribution. Tetramodal models failed to optimize. The bimodal models vary only slightly from a single-gene Mendelian model of 1∶3 distribution. Imposing an exact 1∶3 ratio on the models results in errors very similar to the unconstrained models. In total, these data are consistent with a single gene autosomal dominant Mendelian trait.
(3.38 MB TIF)
