# Supplementary figures and images for: PECAM-Independent Thioglycollate Peritonitis Is Associated With a Locus on Murine Chromosome 2
Source: PLoS One. 2009 Jan 30;4(1):e4316. doi: 10.1371/journal.pone.0004316 (PMC2628736; doi:10.1371/journal.pone.0004316)

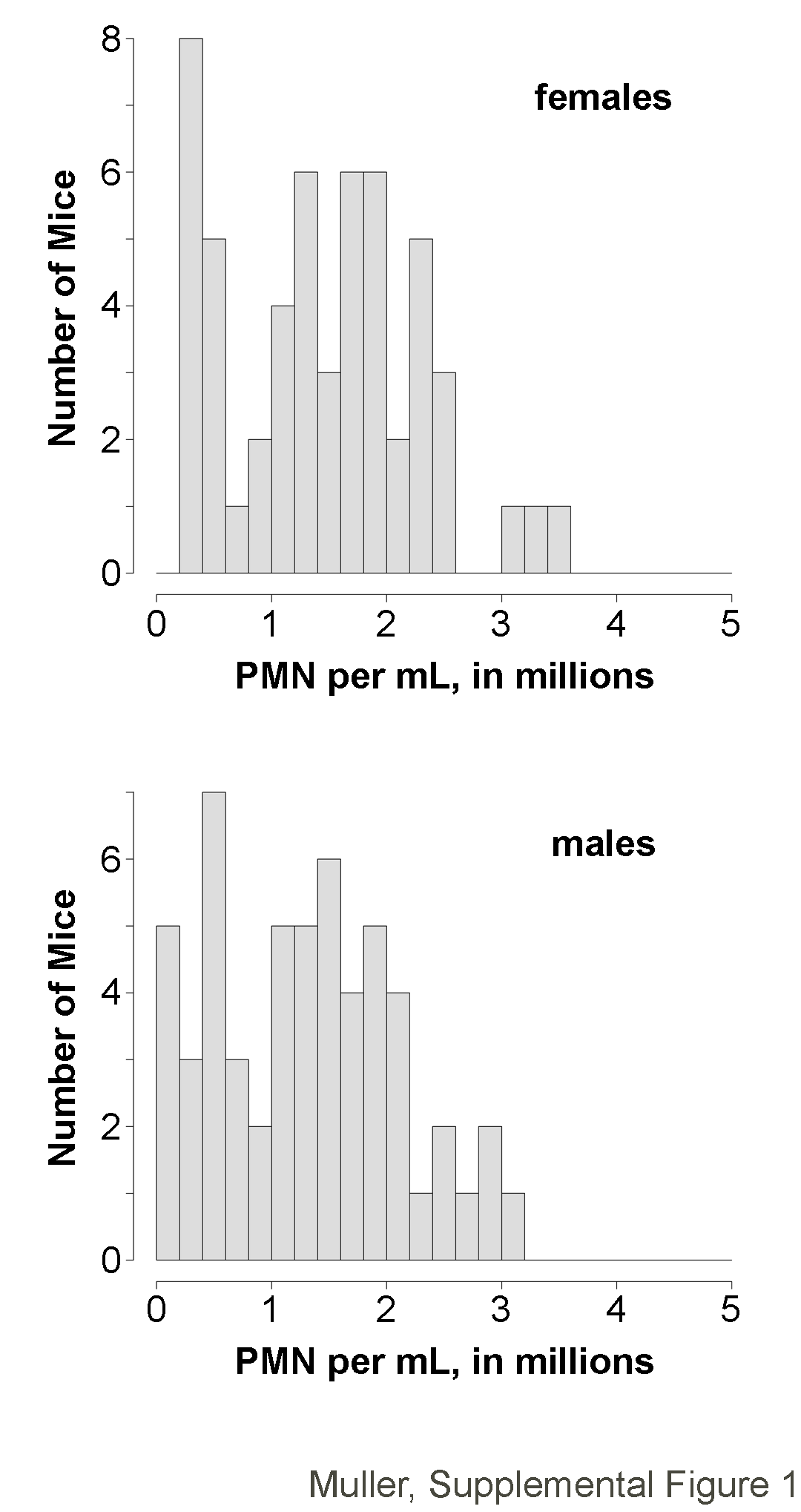

Supplement: Figure S1 — Histograms comparing the distribution of female and male mice in the F2 generation. The histograms each show the number of mice of the given gender having inflammation scores within a given range (cell increment 2×10^5^ PMN per mL). Statistical comparison of the two groups yields a Welch two-sample t-test p-value of 0.296 and a Mann-Whitney (Wilcoxon) u-test p-value of 0.283. (6.63 MB TIF) [file pone.0004316.s002.tif]

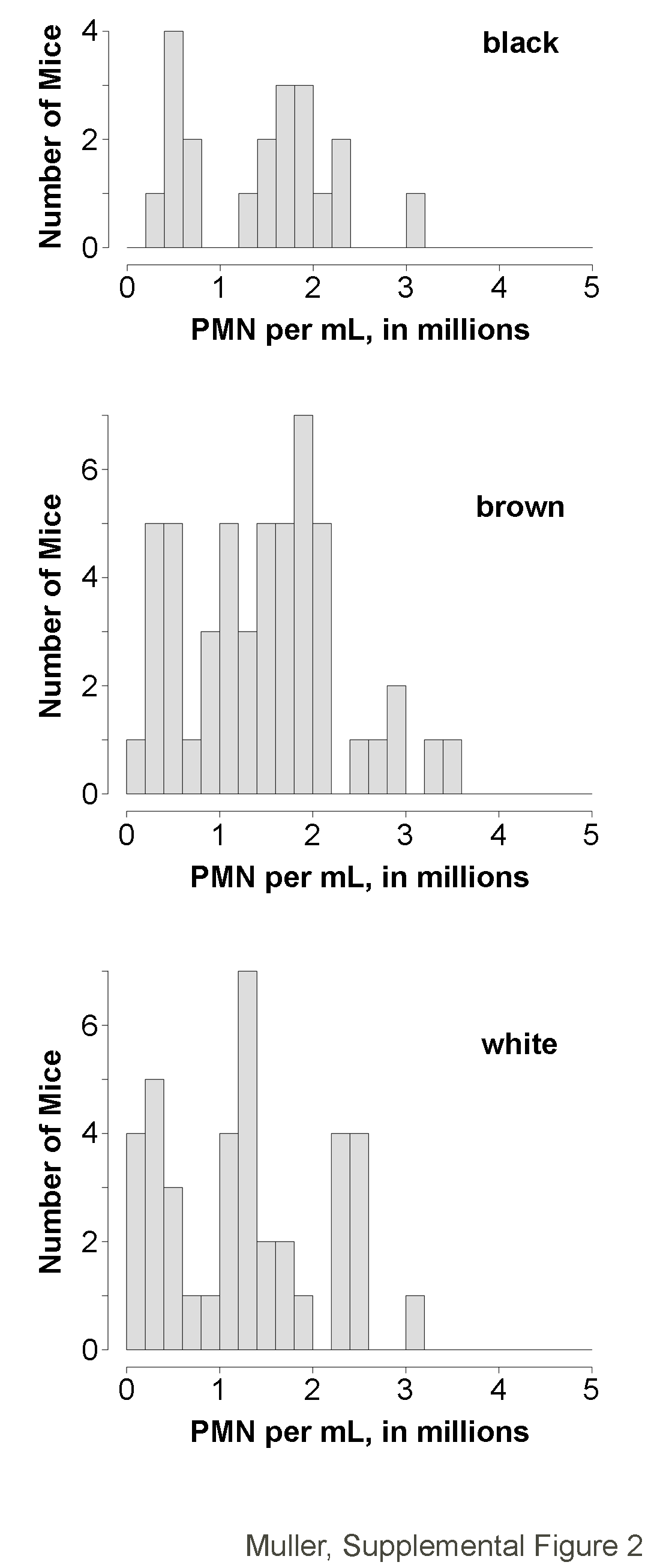

Supplement: Figure S2 — Histograms comparing the distribution of black, brown, and white mice in the F2 generation. The histograms each show the number of mice of the given coat color having inflammation scores within a given range (cell increment 2×10^5^ PMN per mL). Statistical comparison of the black and brown groups yields a Welch two-sample t-test p-value of 0.750 and a Mann-Whitney (Wilcoxon) u-test p-value of 0.924. Statistical comparison of the black and white groups yields a Welch two-sample t-test p-value of 0.515 and a Mann-Whitney (Wilcoxon) u-test p-value of 0.386. Statistical comparison of the brown and white groups yields a Welch two-sample t-test p-value of 0.225 and a Mann-Whitney (Wilcoxon) u-test p-value of 0.204. (8.49 MB TIF) [file pone.0004316.s003.tif]

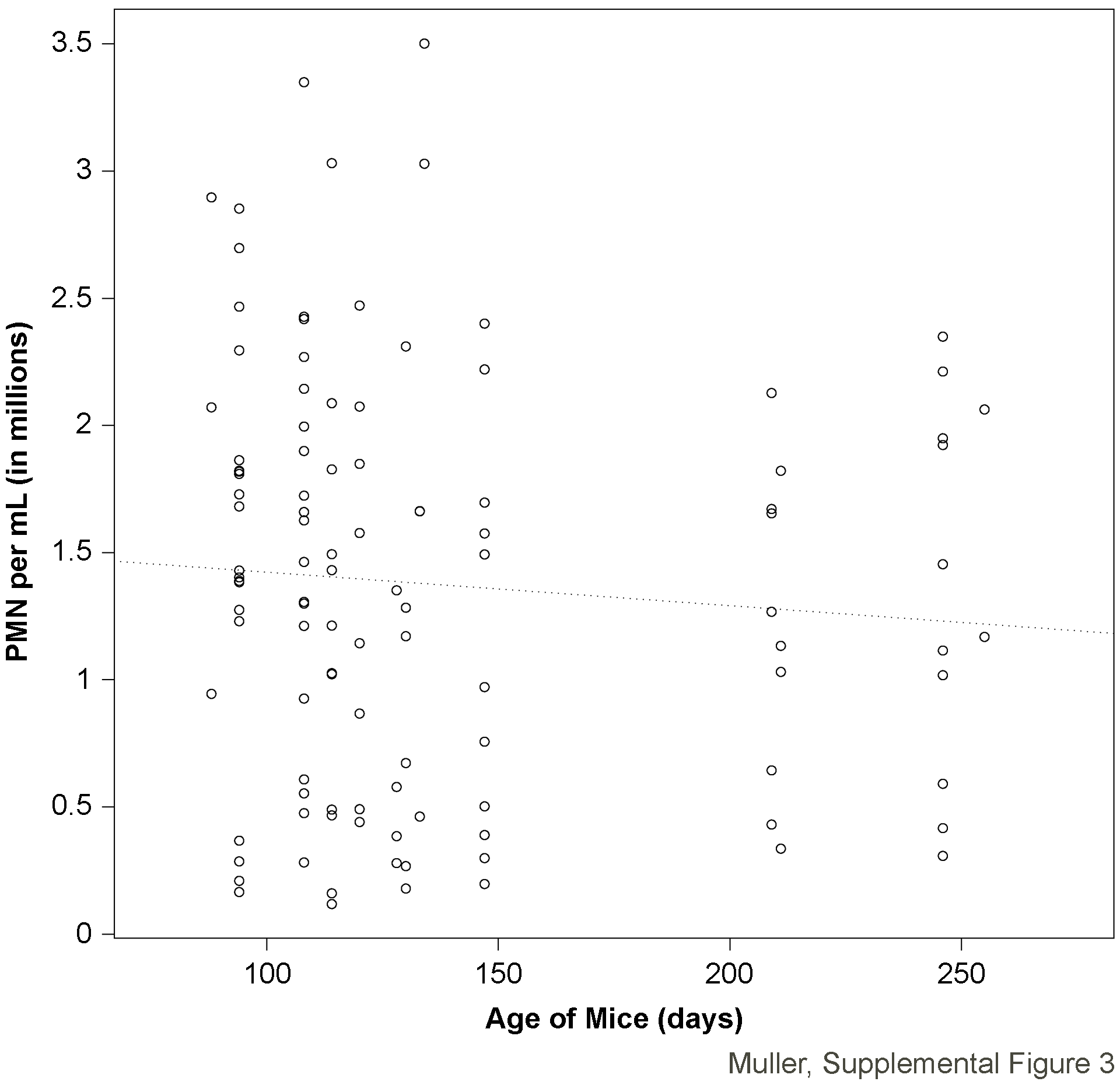

Supplement: Figure S3 — Scatterplot comparing the inflammation scores mice in the F2 generation to the age of the mouse at the time of the experiment. Each circle on the plot represents a single mouse. The dashed line represents a least-squares regression fit; R2 value = 0.002, p-value 0.398. (10.74 MB TIF) [file pone.0004316.s004.tif]

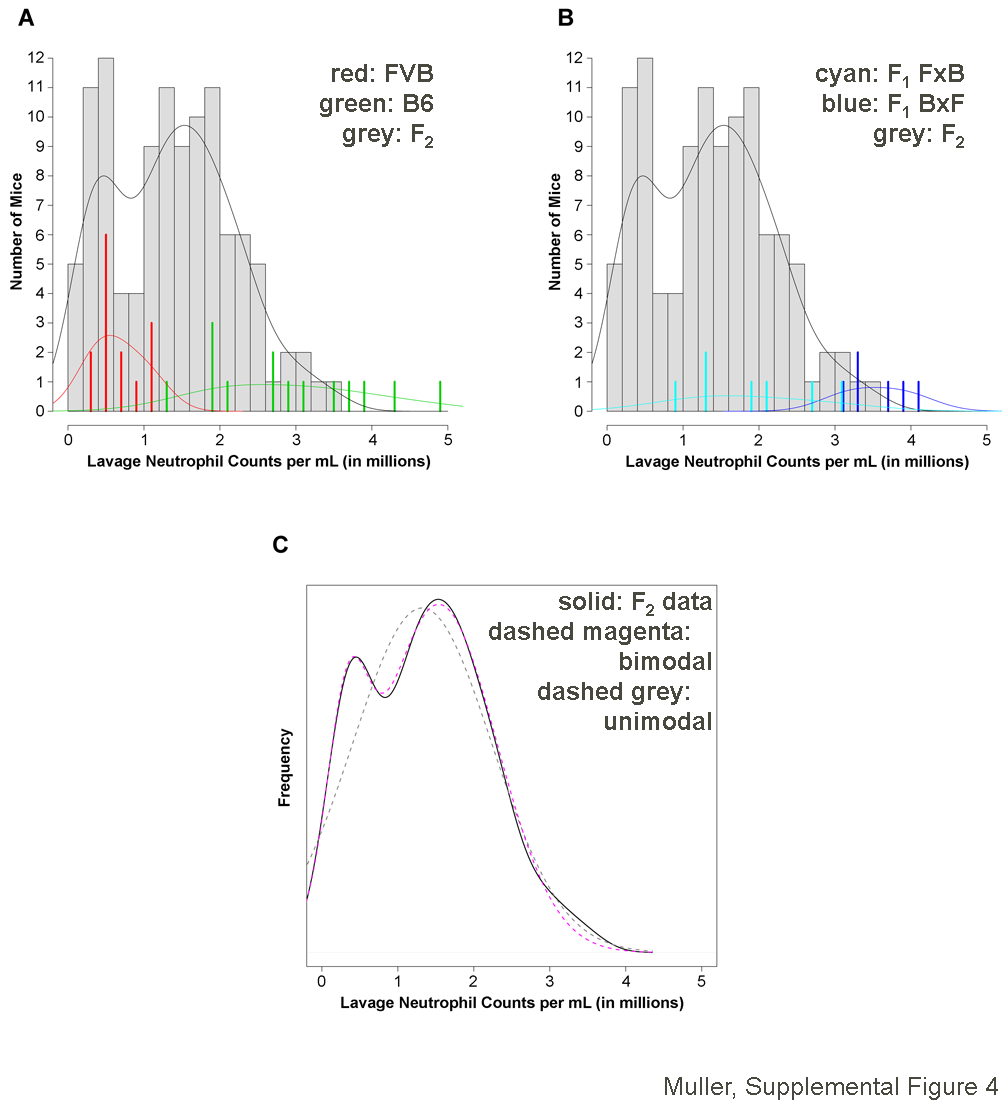

Supplement: Figure S4 — Statistical modelling of the F2 population. Statistical models representing the mathematical distribution of the F2 mice were built in the R statistical package to evaluate for the number of subpopulation that could be identified. Non-parametric densities of the various distributions were estimated using three different functions, density, bkde (at two different kernel values), and hist. The four resulting probability estimates were modeled with the non-linear modeling function, nls, using varying numbers of normally distributed subpopulations. Coefficients were tested against expectations using chi-square and Fisher's exact tests (both tests designed to compare expected and observed count data), and model errors were the residual summed square values (an algorithm that computes the difference between the model and the actual data at hundreds of data points, and returns an unsigned sum of the differences). A representative non-parametric density model is shown overlaying the parental, F1, and F2 populations in panels A and B. Panel C shows the resulting bimodal and unimodal mathematical models, demonstrating that a bimodal distribution, i.e. a mathematical model composed of two overlapping but distinct subpopulations, is more robust than a unimodal model, i.e. a mathematical model in which the mice all appear to be part of a single large population. Trimodal models, not shown, were only subtle variations on the bimodal models, rather than a distinctly different distribution. Tetramodal models failed to optimize. The bimodal models vary only slightly from a single-gene Mendelian model of 1∶3 distribution. Imposing an exact 1∶3 ratio on the models results in errors very similar to the unconstrained models. In total, these data are consistent with a single gene autosomal dominant Mendelian trait. (3.38 MB TIF) [file pone.0004316.s005.tif]
